# Role of climate variability in the potential predictability of tropical cyclone formation in tropical and subtropical western North Pacific Ocean

**DOI:** 10.1038/s41598-019-56243-y

**Published:** 2019-12-27

**Authors:** Yu-Lin K. Chang, Yasumasa Miyazawa, Swadhin Behera

**Affiliations:** Application Laboratory, Japan Agency for Marine-Earth Science and Technology, Yokohama, 236-0001 Japan

**Keywords:** Climate and Earth system modelling, Projection and prediction, Natural hazards

## Abstract

The out of phase tropical cyclone (TC) formation in the subtropical and tropical western North Pacific associated with local low-level wind vorticity anomaly, driven by the remote central and eastern equatorial Pacific warming/cooling, is investigated based on the reanalysis and observational data in the period of 1979−2017. TC frequencies in the subtropical and tropical western North Pacific appear to be connected to different remote heating/cooling sources and are linked to eastern and central Pacific warming/cooling, which are in turn related to canonical El Niño/Southern Oscillation (ENSO) and ENSO Modoki, respectively. TCs formed in subtropics (SfTC) are generally found to be associated with a dipole in wind vorticity anomaly, which is driven by the tropical eastern Pacific warming/cooling. Tropically formed TCs (TfTC) are seen to be triggered by the single-core of wind vorticity anomaly locally associated with the warming/cooling of central and eastern Pacific. The predicted ENSOs and ENSO Modokis, therefore, provide a potential source of seasonal predictability for SfTC and TfTC frequencies.

## Introduction

Tropical cyclones (TCs), one of the major natural disasters in the world, are responsible for a huge loss of lives and extensive damages to properties due to the strong winds, heavy rainfall, and storm surges^1^. The western North Pacific, the most active region for TCs in the world, witness about 26 TCs in a year, accounting for more than 30% of global TCs^2^. The interannual variation in TC formation is often linked to the basin-scale phenomena of the Pacific Ocean. TC activity over the western North Pacific and its potential connection to El Niño and Southern Oscillation (ENSO) phenomenon^3^ has been extensively addressed in the previous studies^4–10^. It is noted that TCs are usually more active during the canonical El Niño years as compared to the La Niña years^6–8^. TC frequency has also been found to be related to El Niño Modoki/La Niña Modoki (ENSO Modoki) phenomenon^11^; anomalously large number of TCs are reported in the western North Pacific during positive ENSO Modoki years^4,5^. While ENSO Modoki relationship is discussed recently, the ENSO variability is widely used in statistical models for seasonal forecasting as it is a leading factor to explain the TC activity in the western Pacific^2,12^. Therefore, realistic prediction of ENSO is treated as an important step for successful TC forecasting using the dynamical climate prediction models^12^.

Although ENSO was considered to influence the TC activity in the western Pacific, individual ENSO indices did not appear to have a high correlation with TC frequency in the entire western North Pacific. For example, the correlation between Niño 3, ENSO Modoki Index (EMI) and TC frequency during 1960−2008 were −0.13 and 0.31, respectively^4^. Similar to this conclusion, a recent study suggested that one index (Niño 3.4 or Niño 4) of ENSO alone did not perform well to prediction TC frequency east of Australia though ENSO phenomenon was a key factor in the prediction. Rather, a combination of multiple tropical climate indices including EMI and Trans-Niño Index yielded better prediction^13^. A previous study indicated that the correlation between canonical ENSO and TC activity in the western North Pacific could be improved by further splitting the formation zones into sub-regions, and they noticed that TC frequency between northern and southern sub-regions was not in phase^4^. Similar results were also reported later on, showing the eastern Pacific warming is associated with more TCs in southeastern sub-region of western North Pacific, whereas fewer TCs were formed in northwestern sub-regions^9,10^. On the other hand, the role of central Pacific warming/cooling in TC formation is not so clear. In some studies, the central Pacific warming/cooling (ENSO Modoki) and western Pacific TC formation were suggested to be significantly correlated^4,5^, while in another study, no significant relation was found between the two^9^. Yet another study suggested that the central Pacific warming is related to TC formation in northwestern sub-region of western North Pacific^10^.

The interannual variation of TC formation is also attributed to the atmosphere-ocean variation. A recent work by Chang *et al*.^14^ found that TC frequencies in subtropical and tropical western North Pacific were out of phase and the TC activity was well correlated to the local dipoles of low-level wind vorticity. The individual ENSO indices alone could not explain well the TC activity in western North Pacific, which could be attributed to the seesaw oscillation of TC formation in subtropical and tropical western North Pacific, that may be forced by multiple mechanisms^14^. Previous studies had hypothesized that wind may play a crucial role in connecting the remote sea surface temperature (SST) in central or eastern Pacific to the TC activity in the western North Pacific Ocean^2,4,15^. ENSO, or the heating of SST in central or eastern Pacific could change the horizontal wind vorticity and vertical wind shear in the western North Pacific thereby influencing TC activity there. Although such relationships are feasible and the participating fields (such as SST, wind and TC frequency) seem to be appropriate, the story remains incomplete. Also, what drives the change of subtropical and tropical wind vorticity and what leads to the out of phase TC formation zones in subtropical and tropical western North Pacific remains unclear. In order to clarify those, the present work aims to investigate the connection between eastern and central Pacific warming/cooling, change of wind vorticity in the western Pacific and the out of phase relationship between subtropical and tropical TC formation zones based on the atmospheric and oceanic reanalysis data. The analysis result is then applied to a statistical TC prediction model based on the forecasts of a coupled climate model to evaluate the predictability skills of the seasonal TC activity in the western North Pacific.

## Data and Methods

Tropical cyclone information from 1945 to 2017 is obtained from the best track data of the Joint Typhoon Warning Center (JTWC, http://www.usno.navy.mil/NOOC/nmfc-ph/RSS/jtwc/best_tracks/). TCs formed in the western North Pacific from 120°−180°E are considered in this study. TCs formed in subtropical and tropical regions are separated by 18°N, which corresponds to the location of zero low-level wind vorticity following our previous study^14^. TCs formed between 18−30°N are defined as subtropically formed TCs (SfTC), whereas those formed between 6−18°N are considered tropically formed TCs (TfTC). Sea surface temperature and wind at 10 m height are from ECMWF Interim dataset at 0.5-degree horizontal resolution for the period from 1979 to present (https://www.ecmwf.int/en/forecasts/datasets/reanalysis-datasets/era-interim). The study period considered for the present work is from 1979 to 2017 that is the common period when all data were available for the analysis. The annual typhoon season in the present work is only considered from June to October. The TC formation location is defined where wind speed exceeds 39 mph and turns into tropical storm following Saffir-Simpson scale. The low-level wind vorticity is computed from the curl of wind at 10 m height (∇ × ***u***), where ***u*** is the wind vector.

Retrospective climate prediction data are provided by the coupled global ocean-atmosphere general circulation model SINTEX-F seasonal prediction system (http://www.jamstec.go.jp/aplinfo/sintexf/e/seasonal/overview2.html)^16–18^. The ensemble system was developed by the Application Laboratory of JAMSTEC, focusing on predicting the tropical climate variation, such as ENSO, ENSO Modoki, the Indian Ocean Dipole, and Indian Ocean Subtropical Dipole etc. The first generation of this system (SINTEX-F1) has performed skillful prediction of ENSO a year ahead, the updated system (SINTEX-F2) further improved the subtropical variability. Results from the latest version SINTEX-F2–3DVAR^18^ is used in the present study. There are 12 ensemble members, the horizontal resolutions are 1.125° and 0.5° for atmospheric and oceanic components, respectively, with 31 levels in the vertical for both. A sea ice model is embedded in the system. The model also assimilates the available *in-situ* ocean temperature and salinity based on the three-dimensional variational ocean data assimilation (3DVAR) scheme. The retrospective prediction data is available from 1983 to 2015. The prospective prediction data is updated in real time once in a month. Global SST dataset was not available for the retrospective predictions, so that 2 m air temperature is used to serve as a proxy of SST. A high correlation is observed between SST and 2 m air temperature of the ECMWF reanalysis data for the study region.

All data are organized into the monthly format and the composite significance is examined by a student t-test. The coupled analysis is computed using singular value decomposition (SVD^19,20^), applied to two fields as it seeks to identity pairs of coupled spatial patterns, with each pair explaining a fraction of covariance between the two fields. The spatial patterns are plotted as homogeneous correlation maps, whose squares give the percentage of variance explained^21^.

## Results

**Figure 1:**
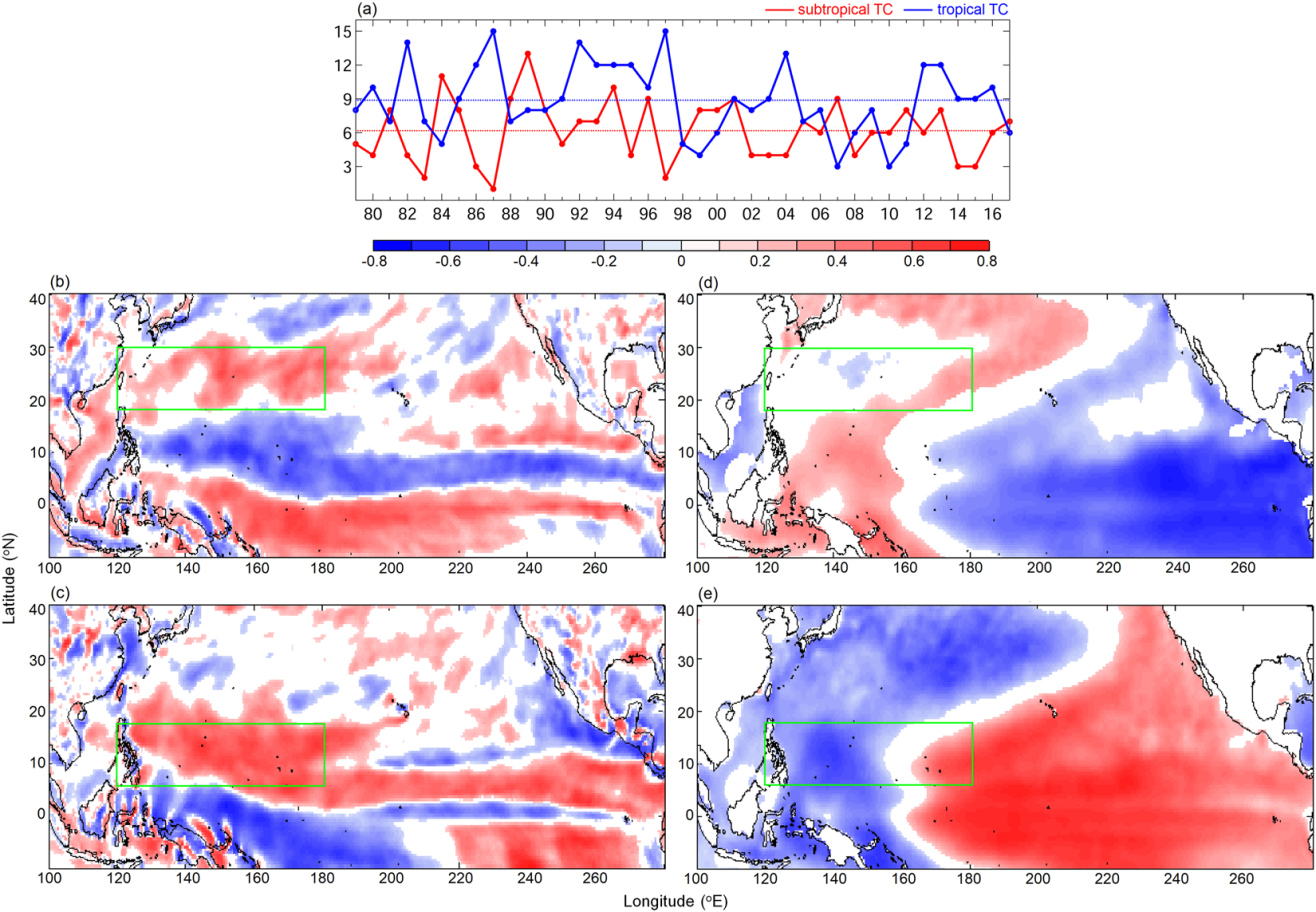
(**a**) Time series of SfTC (blue) and TfTC (red) frequencies. Correlation between TC frequencies and (**b**,**c**) 10 m height wind vorticity anomaly, and with (**d**,**e**) SSTA from ECMWF reanalysis. TC frequencies are from (**b**,**d**) SfTCs and (**c**,**e**) TfTCs. Blue and red lines in (**a**) show the mean TfTCs and SfTCs frequencies, respectively. Values below 95% significance level are masked in white.

The average number of TCs formed in subtropical and tropical western North Pacific during June-October of the study period are 6.2 and 8.9, respectively. TCs formed in subtropical and tropical western North Pacific are generally out of phase, and are negatively correlated (Fig. 1a, r = −0.38, p < 0.05). Our previous study suggested that TC formed in both subtropical and tropical western North Pacific are well correlated with Philippines−Taiwan Oscillations (PTO^22^) which is defined by the dipoles of subtropical and tropical wind vorticity in the western North Pacific^14^. So, the correlation between the low-level wind vorticity and TC frequency in subtropical and tropical western North Pacific is first evaluated (Fig. 1b-e). Interestingly, the correlation coefficient shows a dipole (Fig. 1b) for the TCs formed in subtropics (hereafter SfTCs) though such a dipole signal is not clearly seen when wind vorticity anomaly is correlated to the corresponding TCs formed in the tropics (hereafter TfTCs, Fig. 1c). The SfTCs correlations are consistent with a previous study that suggested low-level wind vorticity distributions in the subtropical and tropical western North Pacific co-oscillate^22^. On the other hand, single-core wind vorticity pattern corresponding to the TfTCs case indicates TC formation in subtropical and tropical western North Pacific may be related to different driving forces.

The difference is extended further when SST anomalies (SSTA) were correlated to the time series of SfTC and TfTC frequencies (Fig. 1d,e). The SfTCs are negatively correlated to SSTA in central to eastern Pacific, especially east of 220°E, which could be associated with canonical ENSO whereas TfTCs are positively correlated to SST in central Pacific between 170°E to 240°E, that may link to ENSO Modoki. In addition, TfTCs and SfTCs seem not so sensitive to local SSTA as TCs are mostly insignificantly correlated to SSTA in the western North Pacific. The high values of absolute SST observed in those regions generally explain the lack of significant positive correlations.

**Figure 2:**
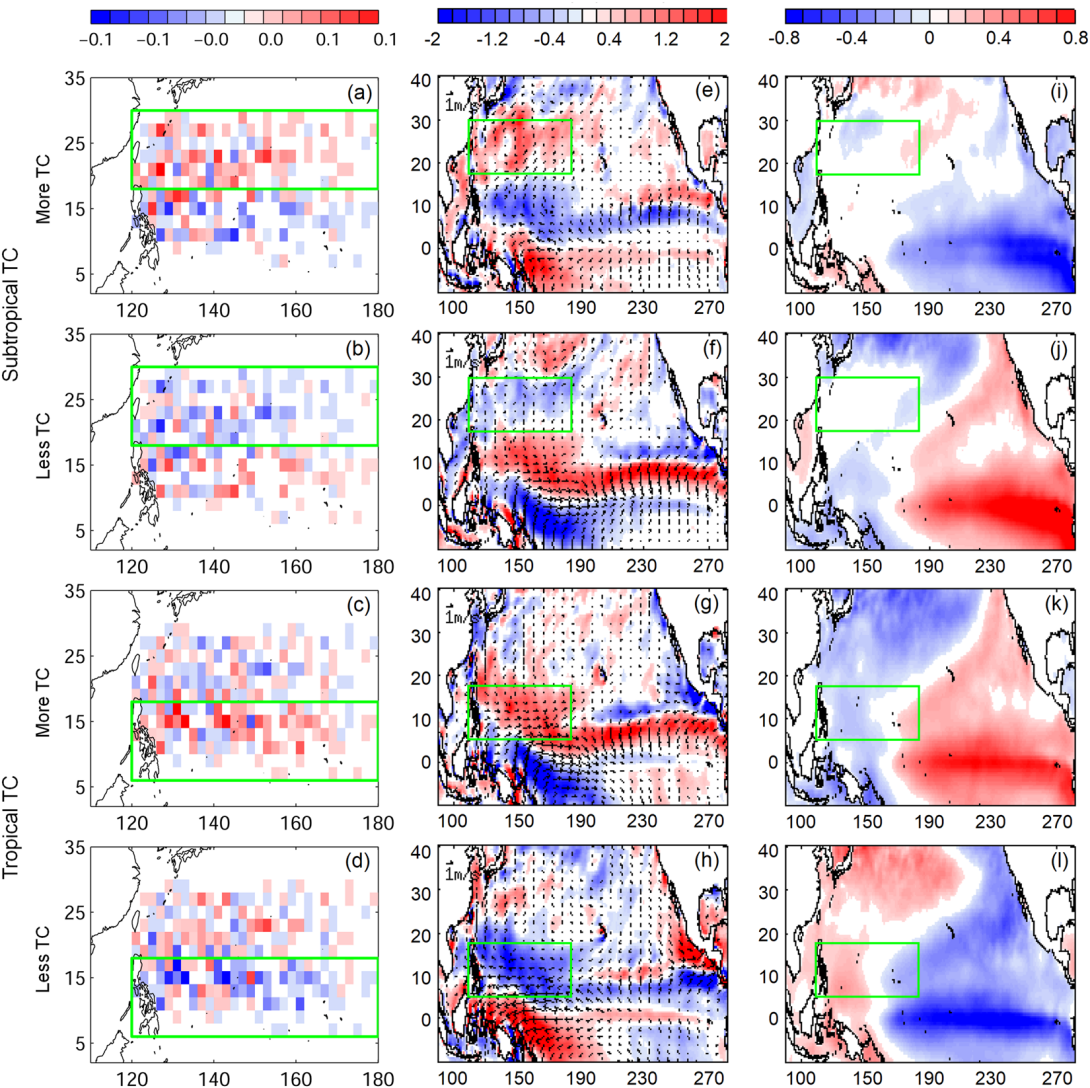
Composites of anomalously greater and fewer SfTC and TfTC periods. (**a**–**d**) TC density anomaly (number of TCs formed per grid per year), (**e**–**h**) 10 m height wind (vector) and vorticity (shading) anomaly, and (**i**–**l**) SSTA. Green boxes indicate the corresponding TCs formation regions. Values below 95% significance level are masked in white.

We have next composited the periods of higher and lower TC activities (based on the average number of TCs shown in Fig. 1a) for SfTCs and TfTCs, respectively. Figure 2 shows the composite patterns of TC density (i.e. number of TCs formed per grid per year) anomaly together with wind vorticity anomaly and SSTA. The out of phase TC formation in tropical and subtropical western North Pacific is also captured in the TC density anomaly map (Fig. 2a–d), though the spatial distributions are sometimes not as organized as the one we saw in TC frequency case (Fig. 1b). Interestingly, the composite based on SfTCs not only show the phase change in subtropical region but also in tropical area. Similarly, the seesaw oscillation between subtropical and tropical regions is seen in the composite of TfTCs. It is also noted that more subtropical TCs are formed during the simultaneous cooling of eastern Pacific SST with positive and negative wind vorticity in the subtropical and tropical western North Pacific, respectively (Fig. 2e,i). The patterns are reversed during the periods of less subtropical TC formations, except when the warming of SST enhances towards central Pacific (Fig. 2f,j). Conversely TfTCs are more when the central to eastern Pacific is warmer, and the positive wind vorticity appears in the tropical western North Pacific (Fig. 2g,k). The ocean-atmosphere conditions are reversed during the low activity period of tropical TCs (Fig. 2h,l).

**Figure 3:**
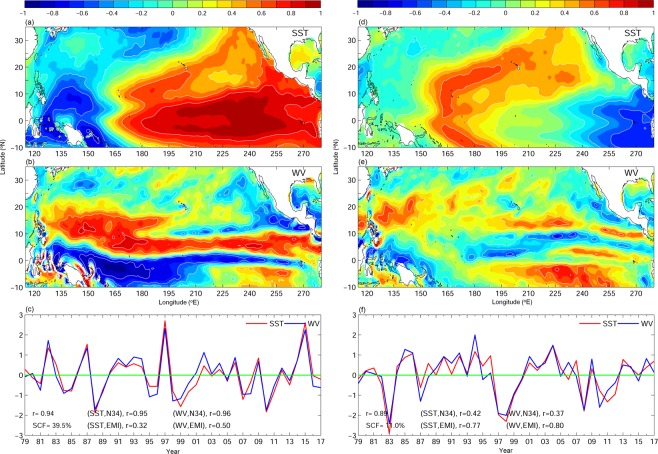
(left) mode 1 and (right) mode 2 of SVD analysis from ECMWF. (**a**,**d**) spatial patterns of SST and (**b**,**e**) 10 m height wind vorticity, and (**c**,**f**) expansion coefficients. SCF stands for squared covariance fraction, r is the coupled coefficient between the two spatial patterns.

The coupled variability between SSTA and wind vorticity anomaly is also found in a SVD analysis. Figure 3 shows the SVD coupled spatial patterns and expansion coefficient between SSTA and low-level wind vorticity anomaly. Mode 1 explains 39.5% of the total variance. It shows that the warming of eastern Pacific SSTA is associated with positive wind vorticity anomaly in the tropical western North Pacific, and slightly weaker negative wind vorticity anomaly in the subtropics (Fig. 3a,b). The two patterns are highly linked with a coupling coefficient of 0.94. However, we noted that expansion coefficients are highly correlated to both Niño 3 and Niño 3.4 index (r > 0.9, p < 0.05) (Fig. 3c). Therefore, canonical ENSO played a significant role in their variability. The SVD mode 2 accounts for 11% of the total variance, showing the associated patterns of a central Pacific warm SSTA and a positive wind vorticity anomaly in the tropical western North Pacific (Fig. 3d,e). The two spatial patterns are well coupled (r = 0.89, p < 0.05), and their expansion coefficients are highly correlated to EMI (r ~ 0.8, p < 0.05), inferring the importance of ENSO Modoki in this case. The connection between warming/cooling of central and eastern Pacific and the different wind vorticity in the western North Pacific has been demonstrated by the previous study based on the numerical experiments^4^. They have shown that canonical El Niño associated with eastern Pacific warming would drive a dipole of low-level wind vorticity, with positive and negative wind vorticities in the tropical and subtropical western North Pacific respectively. On the contrary, the central Pacific warming by El Niño Modoki triggered a single-core of positive vorticity in the tropical western North Pacific. Similar patterns of coupled signals between SST and low-level wind vorticity are also found in the past 39-years of reanalysis data using SVD analysis in the present study. The SVD mode 1 spatial patterns are similar to the composite maps based on SfTCs (Figs. 2 and 3). The mode 1 time series is significantly correlated to SfTCs frequency (r = −0.62, p < 0.05) but it is also related to TfTCs (r = 0.64, p < 0.05). On the other hand, the mode 2 wind vorticity resembles the composite maps for TfTCs (Figs. 2 and 3), showing a single core of wind vorticity anomaly in the tropical western Pacific. However, discrepancy is seen in SSTA. Mode 2 SVD and TfTCs composite both capture the central Pacific warming/cooling, but the variations in eastern Pacific are different between the two. In spite of this general dissimilarity in the eastern Pacific associated with them, the mode 2 SVD has a significant correlation with TfTCs (r = 0.39, p < 0.05) as well as SfTCs (r = −0.38, p < 0.05).

The SVD results bring out the leading roles of ENSO and ENSO Modoki in driving the wind vorticity anomaly in the western North Pacific. The teleconnection mechanism between SST warming/cooling and wind vorticity could be a Matsuno-Gill response^23,24^. Further, the significant correlation between SVD and TC frequencies indicates the change of wind vorticity may further influence TCs formation in the subtropical and tropical western North Pacific. Although the composite of low-level wind vorticity anomaly clearly shows the contrasting signals between the subtropical and tropical formed TC frequencies, and also between the TC rich and poor periods, the corresponding SSTA composite is less distinct (Fig. 2). SfTCs is related to warming/cooling in the eastern Pacific, corresponding to canonical ENSO (Figs. 1–3) variability. On the other hand, the TfTCs are associated with eastern Pacific warming/cooling as well as that of central Pacific (Figs. 1 and 2). Therefore, those could be influenced by both canonical ENSO as well as ENSO Modoki. Although both subtropical and tropical TC frequencies significantly correlate to the leading modes of ENSO and ENSO Modoki picked up by the SVD analysis, the subtropical western Pacific wind vorticity anomaly is not induced by the central Pacific warming/cooling (Figs. 2 and 3). Therefore, ENSO Modoki does not seem to play a major role in affecting the subtropical TC formation. On the other hand, the composite SST anomaly for TfTCs (Fig. 2g,h) shows the warming/cooling is not necessarily confined to central Pacific, but also extends to eastern Pacific. Indeed, the canonical El Niño year and El Niño Modoki year could sometimes overlap. Not only the central Pacific warming, the eastern Pacific warming during El Niño year did also drive a positive wind vorticity in the tropical western Pacific (Figs. 2 and 3). Therefore, the TfTCs could be influenced by both canonical ENSO and ENSO Modoki.

**Figure 4:**
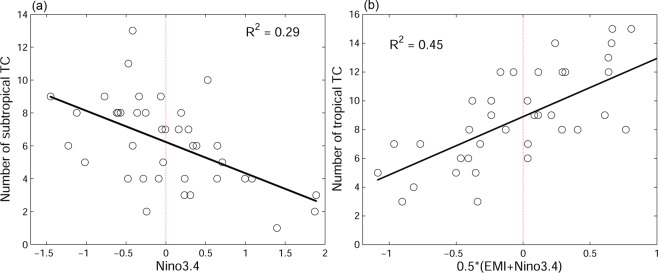
Regression (**a**) between subtropical TCs and Niño 3.4 index, and (**b**) between tropical TCs and Niño 3.4 together with ENSO Modoki index.

In order to confirm the relation between canonical ENSO and SfTC frequency, as well as between canonical ENSO/ENSO Modoki and TfTC frequency, we regress numbers of TfTCs and SfTCs to Niño 3.4 Index and EMI (Fig. 4). The SfTCs time series is negatively correlated to the Niño 3.4 Index (r = −0.54, p < 0.05), and the derived linear regression formula is;

$$T{C}_{subtropics}=6.23-1.9\times Nino3.4$$

On the other hand, the number of TfTCs is positively correlated to EMI (r = 0.48, p < 0.05) as well as Niño 3.4 Index (r = 0.65, p < 0.05). Therefore, we apply the regression by taking into the consideration of both Niño 3.4 Index and EMI, and derive the best fit to tropical formed TC frequency. The best fit index turns out to be equally weighted for Niño 3.4 and EMI, with a higher correlation to TfTCs (r = 0.68, p < 0.05). The corresponding regression formula is;

$$T{C}_{tropics}=8.9+4.04\times 0.5(Nino3.4+EMI)$$

The TfTCs are collectively influenced by canonical ENSOs and ENSO Modokis. Also, the influences are seen to be a bit asymmetric depending on the phase of both phenomena. The period of fewer TfTCs seems to have greater connection to La Niña Modoki as compared to a similar connection between higher TfTCs and El Niño Modoki. Similarly, higher TfTCs are better connected to El Niño as compared to the opposite connection between lower number of TfTCs and La Niña (Fig. 4b). ENSO Modoki, however, does not seem to influence SfTCs, as the central Pacific warming/cooling does not drive the subtropical wind vorticity. Therefore, the correlation between SfTC frequency and EMI is insignificant (r = 0.02, p > 0.9).

**Figure 5:**
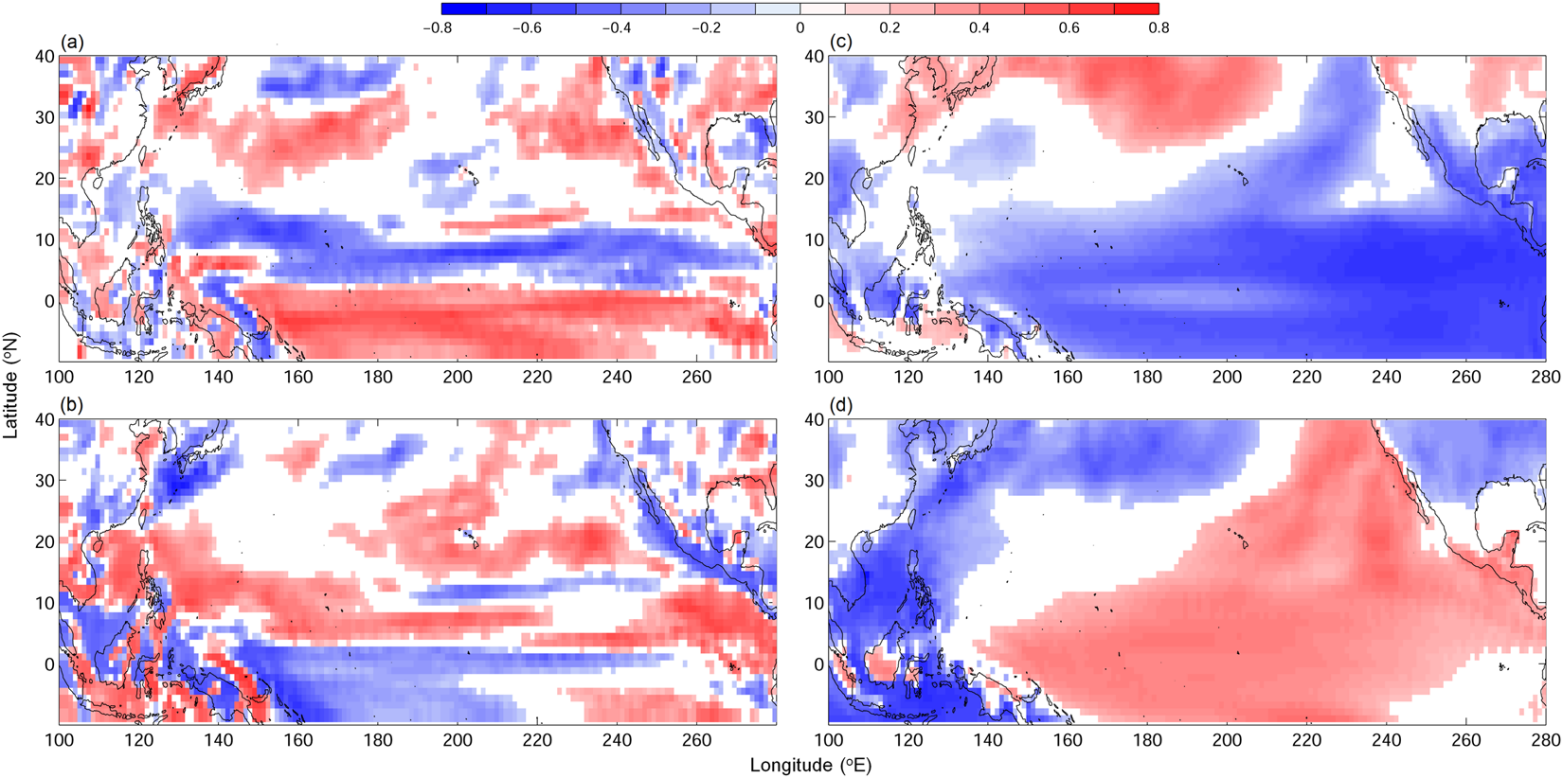
Correlation between TC frequencies and (**a**,**b**) low-level wind vorticity anomaly at 1000 mb, and with (**c**,**d**) 2-meter air temperature from retrospective forecasting model SINTEX-F. TC frequencies are from (**a**,**c**) SfTCs, and (**b**,**d**) TfTCs. Values below 95% significance level are masked in white.

**Figure 6:**
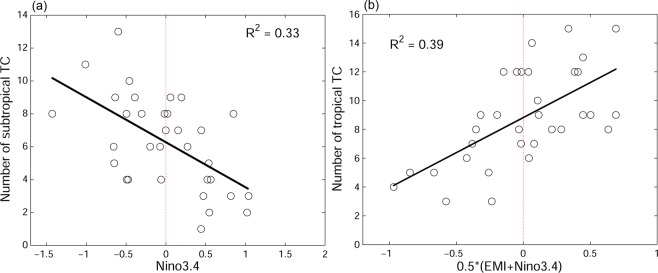
Same as Fig. 4 but ENSO/ENSO Modoki indies are from SINTEX-F hindcast prediction.

For potential future prediction using the seasonal forecast system, the capability of retrospective prediction from SINTEX-F is examined. The analyses of retrospective forecast wind vorticity anomaly and 2 m air temperature reveal similar correlations to SfTCs and TfTCs as already seen in the reanalysis data (Figs. 1 and 5); showing SfTCs are related to dipole wind vorticity anomaly associated with eastern Pacific warming/cooling, whereas TfTCs are related to tropical wind vorticity anomaly driven by central to eastern Pacific warming/cooling. Moreover, the predicted Niño 3.4 and EMI from SINTEX-F hindcast are found to be well correlated to SfTCs and TfTCs (r ~ 0.6, Fig. 6), and the relation to TCs frequencies are comparable to reanalysis ENSO/ENSO Modoki indices (Figs. 4 and 6). The results suggest the feasibility of the prediction system. Owing to the relation between ENSO/ENSO Modoki indices and SfTCs and TfTCs, the well-predicted ENSO/ENSO Modoki indices could therefore provide a potential source of seasonal predictability to further predict TC frequencies in the subtropical and tropical western North Pacific.

## Summary and Discussion

The present study explores the out of phase TC formations in subtropical and tropical western North Pacific in relation to central and eastern equatorial Pacific warming/cooling based on the past 39 years (1979–2017) atmospheric and oceanic reanalysis data and observations. More TCs were generated over the positive low-level wind vorticity anomaly region. The observed seesaw oscillation between subtropical and tropical wind vorticity^14,22^ may explain the asynchronous TC formations in subtropical and tropical western North Pacific. Frequencies of TCs in the subtropical and tropical western North Pacific appear to be connected to different remote forcings that are generally linked to canonical ENSO and the combined effect of canonical ENSO and ENSO Modoki, respectively. The cooling of the eastern Pacific (La Niña) drives the dipole of low-level wind vorticity anomaly in the western North Pacific. The northern pole of positive wind vorticity in subtropical western North Pacific is associated with more TC formations there. Conversely, negative wind vorticity anomaly driven by warming of eastern Pacific (El Niño) is associated with fewer SfTCs. On the other hand, more TCs form in the tropical western North Pacific when the central and eastern Pacific warms up (owing to El Niño Modoki/El Niño) that drives a positive wind vorticity anomaly in the tropical western North Pacific and provides a favorable condition for TC formation. The condition is reversed during opposite phase (La Niña Modoki/ La Niña) years, which results in negative wind vorticity anomaly in tropical western North Pacific leading to fewer TC formations there.

Previous studies suggested that TCs were more active during the canonical El Niño years as compared to the La Niña years^6,7^. However, a weak negative correlation between canonical ENSO and western Pacific TC activity was suggested by another work^4^. The spatial variance of TC formation in the western North Pacific had been noticed^4,6^, and a recent study further suggested the out of phase TC activity in the subtropical and tropical regions^14^. By separating TCs formed in subtropical and tropical western North Pacific, we further clarify here that canonical El Niño years are associated with less TC formations in the subtropical region and formations of more TCs in the tropical western North Pacific. Similar results were also pointed out in a previous study^4^, in which they found a negative correlation between Niño 3 and TCs formed north of 15°N, and a positive correlation with TCs formed south of 15°N and east of 150°E. Apart from canonical ENSO, ENSO Modoki was also found to be significantly correlated with western North Pacific TC frequency, showing more TCs during El Niño Modoki years^4,5^. A similar tendency is also noticed in the present study. However, the significant correlation only appears for the TCs in the tropical western North Pacific but not in the subtropical region.

The previous study proposed the local climate index PTO could serve as a promising proxy in explaining TC activity and have also shown that PTO performed a better correlation than individual ENSO indices^14^. PTO, defined by the dipole of wind vorticity that reveals seesaw oscillation between subtropical and tropical western North Pacific could potentially be well related to out of phase of SfTCs and TfTCs. The local wind vorticity indeed plays an important role in affecting TC formation. However, the dipole wind vorticity is only related to SfTCs, but not TfTCs (Fig. 1). Therefore, instead of PTO, the local low-level wind vorticity or the corresponding remote forcing (central/eastern Pacific warming/cooling), have better potentials for the predictability of SfTC as well as TfTC frequencies.

Seasonal prediction system SINTEX-F predicts Nino indices reliably and is useful in the prediction of SfTC and TfTC frequencies in the western North Pacific. A direct comparison of wind vorticity and formed tropical cyclones in the model would have served as a better evaluator of our argument. However, the seasonal forecast system does not have enough resolutions yet to fully resolve the tropical cyclone frequencies due to limited computing resources. Improving the spatial and temporal resolution for long-term predicting model would be our future goal.

## References

[1] Shultz JM, Russell J, Espinel Z (2005). Epidemiology of Tropical Cyclones: The Dynamics of Disaster, Disease, and Development. Epidemiologic Reviews.

[2] Landsea, C. W. *Climate variability of tropical cyclones: Past*, *Present and Future* 220–241 (2000).

[3] Philander, S. G. *El Niño, La Niña, and the Southern Oscillation*. (Academic Press 1989).

[4] Chen Guanghua, Tam Chi-Yung (2010). Different impacts of two kinds of Pacific Ocean warming on tropical cyclone frequency over the western North Pacific. Geophysical Research Letters.

[5] Pradhan, P. K., Preethi, B., Ashok, K., Krishnan, R. & Sahai, A. K. Modoki, Indian Ocean Dipole, and western North Pacific typhoons: Possible implications for extreme events. *Journal of Geophysical Research: Atmospheres* **116**, 10.1029/2011JD015666 (2011).

[6] Chan JCL, Liu KS (2004). Global Warming and Western North Pacific Typhoon Activity from an Observational Perspective. Journal of Climate.

[7] Chan Johnny C. L. (2000). Tropical Cyclone Activity over the Western North Pacific Associated with El Niño and La Niña Events. Journal of Climate.

[8] Wang, B. & Chan, J. C. L. How Strong ENSO Events Affect Tropical Storm Activity over the Western North Pacific. *Journal of Climate* **15**, 1643–1658, 10.1175/1520-0442(2002)015<1643:hseeat>2.0.co;2 (2002).

[9] Wang C, Li C, Mu M, Duan W (2013). Seasonal modulations of different impacts of two types of ENSO events on tropical cyclone activity in the western North Pacific. Climate Dynamics.

[10] Kim H-M, Webster PJ, Curry JA (2011). Modulation of North Pacific Tropical Cyclone Activity by Three Phases of ENSO. Journal of Climate.

[11] Ashok, K., Behera, S. K., Rao, S. A., Weng, H. & Yamagata, T. El Niño Modoki and its possible teleconnection. *Journal of Geophysical Research: Oceans* **112**, 10.1029/2006JC003798 (2007).

[12] Camargo, S. G., Barnston, A., Klotzbach, P. & Landsea, C. *Seasonal tropical cyclone forecasts*. Vol. 56 (2007).

[13] Ramsay HA, Richman MB, Leslie LM (2014). Seasonal Tropical Cyclone Predictions Using Optimized Combinations of ENSO Regions: Application to the Coral Sea Basin. Journal of Climate.

[14] Chang Y-LK, Miyazawa Y, Kodaira T, Behera S (2018). Philippines–Taiwan Oscillations and its connection to tropical cyclone frequency in the western North Pacific Ocean. Scientific Reports.

[15] Matsuura T, Yumoto M, Iizuka S (2003). A mechanism of interdecadal variability of tropical cyclone activity over the western North Pacific. Climate Dynamics.

[16] Luo J-J, Masson S, Behera S, Shingu S, Yamagata T (2005). Seasonal Climate Predictability in a Coupled OAGCM Using a Different Approach for Ensemble Forecasts. Journal of Climate.

[17] Doi T, Behera SK, Yamagata T (2016). Improved seasonal prediction using the SINTEX-F2 coupled model. Journal of Advances in Modeling Earth Systems.

[18] Doi T, Storto A, Behera SK, Navarra A, Yamagata T (2017). Improved Prediction of the Indian Ocean Dipole Mode by Use of Subsurface Ocean Observations. Journal of Climate.

[19] Bretherton, C. S., Smith, C. & Wallace, J. M. An Intercomparison of Methods for Finding Coupled Patterns in Climate Data. *Journal of Climate* 5, 541–560, 10.1175/1520-0442(1992)005<0541:aiomff>2.0.co;2 (1992).

[20] Wang, D.-P., Oey, L.-Y., Ezer, T. & Hamilton, P. Near-Surface Currents in DeSoto Canyon (1997–99): Comparison of Current Meters, Satellite Observation, and Model Simulation. *Journal of Physical Oceanography* **33**, 313–326, 10.1175/1520-0485(2003)033<0313:nscidc>2.0.co;2 (2003).

[21] Chang Y-L, Oey L-Y (2013). Coupled Response of the Trade Wind, SST Gradient, and SST in the Caribbean Sea, and the Potential Impact on Loop Current’s Interannual Variability. Journal of Physical Oceanography.

[22] Chang Y-L, Oey L-Y (2012). The Philippines–Taiwan Oscillation: Monsoonlike Interannual Oscillation of the Subtropical–Tropical Western North Pacific Wind System and Its Impact on the Ocean. Journal of Climate.

[23] Matsuno T (1966). Quasi-Geostrophic Motions in the Equatorial Area. Journal of the Meteorological Society of Japan. Ser. II.

[24] Gill AE (1980). Some simple solutions for heat‐induced tropical circulation. Quarterly Journal of the Royal Meteorological Society.

